# Supplementary material for: Association between oral microbiome and seven types of cancers in East Asian population: a two-sample Mendelian randomization analysis
Source: Front Mol Biosci. 2023 Nov 21;10:1327893. doi: 10.3389/fmolb.2023.1327893 (PMC10702768; doi:10.3389/fmolb.2023.1327893)
Supplement: Supplementary file 1 [file DataSheet1.ZIP › Supplementary Materials/MR plots for tongue/tongue═╝/Prostate cancer/pheno.1371_to_prostate cancer_forest.pdf]

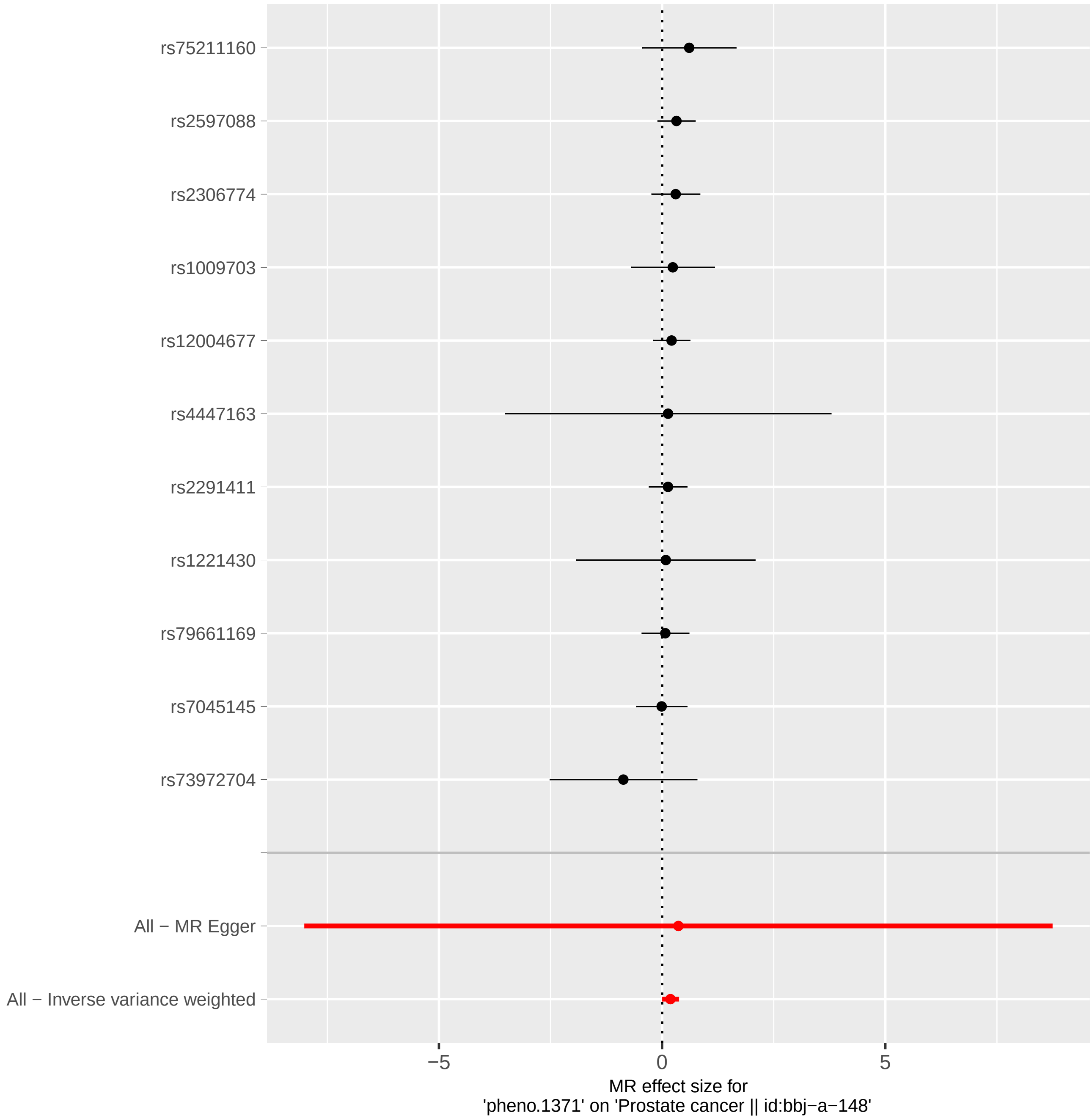
rs75211160
rs2597088
rs2306774
rs1009703
rs12004677
rs4447163
rs2291411
rs1221430
rs79661169
rs7045145
rs73972704
All − MR Egger
All − Inverse variance weighted
−5
0
5
MR effect size for
'pheno.1371' on 'Prostate cancer || id:bbj−a−148'
